# Disability and quality of life in patients with fibromyalgia

**DOI:** 10.1186/1477-7525-6-8

**Published:** 2008-01-22

**Authors:** Jeanine A Verbunt, Dia HFM Pernot, Rob JEM Smeets

**Affiliations:** 1 Rehabilitation Foundation Limburg, P.O. Box 88, 6430 AB Hoensbroek, The Netherlands; 2 Department of Psychological Science, Maastricht University, P.O. Box 616, 6200 MD Maastricht, The Netherlands; 3 Department of General Practice, Maastricht University, P.O. Box 616, 6200 MD Maastricht, The Netherlands; 4 Laurentius Hospital, Mgr.Driessenstraat 6, 6043 CV Roermond, The Netherlands; 5 Blixembosch Rehabilitation Centre, P. O. Box 1355, 5602 BJ Eindhoven, The Netherlands

## Abstract

**Background:**

Patients with fibromyalgia often feel disabled in the performance of daily activities. Psychological factors seem to play a pronounced disabling role in fibromyalgia.

The objectives of the study are: Firstly, to investigate contributing factors for disability in fibromyalgia. Secondly, to study psychological distress in patients with fibromyalgia as compared to other nonspecific pain syndromes. And finally, to explore the impact of fibromyalgia on a patient's quality of life.

**Methods:**

In this cross sectional study, explaining factors for disability were studied based on a regression analysis with gender, mental health, physical and social functioning as independent variables. For the assessment of disability in fibromyalgia the FIQ was used. The levels of psychological distress in patients with fibromyalgia, Complex Regional Pain Syndrome (CRPS) and chronic low back pain (CLBP) were compared based on scores on the Symptom Checklist (SCL90). Quality of life of patients with fibromyalgia was compared with scores (SF36) of both patients with fibromyalgia and other health conditions as derived from the literature.

**Results:**

Disability in fibromyalgia seemed best explained by a patients mental health condition (β = -0.360 p = 0.02). The level of psychological distress was higher in patients with fibromyalgia as compared to patients with CRPS or CLBP (p < 0.01). The impact of fibromyalgia on quality of life appeared to be high as compared to the impact of other health conditions.

**Conclusion:**

Patients with fibromyalgia report a considerable impact on their quality of life and their perceived disability level seems influenced by their mental health condition. In comparison with patients with other pain conditions psychological distress is higher.

## Background

Musculoskeletal diseases are a major public health problem in western society with a high impact on both health care and total societal costs [1]. 41% of the male and even 48% of the female Dutch population aged over 25 years reported to have at least one musculoskeletal disease [2]. Within this survey, localized pain problems, such as "tendinitis" or "capsulitis" were most frequently reported. Fortunately, the impact of these localized pain problems on a patient's quality of life appeared to be only limited as compared with the impact of other pain problems [3]. In contrast with this, fibromyalgia, a pain syndrome characterised by widespread muscle pain, was associated with the highest impact on daily life [3]. Since, the underlying mechanism of fibromyalgia is still unidentified, its especially challenging to find out what makes that patients with fibromyalgia feel disabled in such a high degree and perceive such a high impact of their health problem on their quality of life.

In the last decennia the focus of research on pain related disability has been shifted from a biomedical view to a holistic perspective in which in addition to biomedical also psychological and social factors have their influence [4]. A prominent explanatory model for pain related disability in which biopsychosocial factors are integrated is the fear-avoidance model [5]. According to this model, catastrophic thoughts about pain may lead to an increase of pain-related fear, which in turn is associated with avoidance behaviour. Depression and disuse (i.e., a state of inactivity) may evolve, which in turn are associated with decreased pain tolerance and a higher level of disability. Although the construct of fear of injury is also applicable in patients with fibromyalgia, the mean score on fear of injury of patients with fibromyalgia is lower and the impact of fear on disability seems less high as compared to the impact of fear in other pain syndromes (such as work-related upper extremity disorders, CLBP, osteoarthritis) [6,7].

In addition to the fear avoidance model, alternative models have been proposed to explain disability in chronic pain. Hasenbring hypothesized that, in addition to patients using avoidance strategies as a coping mechanism, other patients with pain will have the tendency to cope with pain using persistent strategies [8]. These patients persist in the performance of activities and appear to ignore their pain and overload their muscles (overuse), resulting in muscular hyperactivity. Long-term muscular hyperactivity can eventually cause chronic pain and long term false straining of the muscles eventually can result in chronification of pain. In accordance with the hypothesis of Hasenbring, Van Houdenhoven suggested that, especially in patients with fibromyalgia and chronic fatigue syndrome, a high level of "action proneness", promoting an overactive lifestyle, may play a predisposing, initiating and/or perpetuating role in the level of disability [9]. According to van Houdenhoven, personality features, such as a high achievement motivation, obsessive-compulsive traits, perfectionism, "workaholism" and self-sacrificing tendencies seem to be related to an overactive lifestyle as a way of coping to prevent anxiety and depression [9]. People who have an overactive lifestyle may run a higher risk of overburdening. If these persons are deprived of overactivity as their favourite coping strategy, for example due to pain or functional limitations, the level of psychological distress can increase. According to van Houdenhoven, especially anxiety and depression seem to have a substantial influence on the level of disability in fibromyalgia.

It seems that several explanatory models for activity related behaviour in musculoskeletal pain might be applicable. McCracken et al confirmed this supposition by the finding that different activity related patterns can be present in patients with chronic pain disability [10]. Based on observations in clinical practice, patients with fibromyalgia seem to present more often persistent behaviour as compared to patients with other nonspecific pain syndromes. As a result, it can be hypothesized that their level of psychological distress will be higher as compared to patients with other pain-syndromes.

The aim of the current study was threefold:

Firstly, the aim of this study was to investigate contributing factors (gender, psychologic, physical and social) to the level of disability in patients with fibromyalgia. Secondly, to study psychological distress in patients with fibromyalgia as compared to patients with other nonspecific pain syndromes. And finally, to explore the impact of the fibromyalgia syndrome on a patient's quality of life as compared to patients with other chronic pain conditions and the general population.

## Methods

### Patients

Patients with fibromyalgia were referred to the study by a consultant in rehabilitation medicine of the department of rehabilitation medicine in one of the five participating hospitals in the South of the Netherlands. They were referred to the department by a medical specialist or general practitioner. Their pain syndrome was labelled as fibromyalgia by a rheumatologist (94.6%) or a general practitioner (5.4%). In order to be able to contrast the impact of fibromyalgia on a patient's daily life situation, patients with two other non-specific pain syndromes were included. Firstly, patients with Complex Regional Pain Syndrome (CRPS) were included in the study; they were referred to the rehabilitation department by one of the anaesthesiologists of the pain clinic in the Laurentius hospital in Roermond. And secondly, patients with chronic low back pain (CLBP) were included who visited a physiatrist in a tertiary care rehabilitation centre after referral by a physiatrist of one of the five before mentioned hospitals. Inclusion criteria were: (1) one of the pain syndromes: fibromyalgia, CRPS or CLBP. (2) no other somatic disease, that could be responsible for the reported pain complaints. (3) sufficient knowledge of the Dutch language in order to be able to read and interpret the questionnaire.

The information and informed consent procedure was approved by the Medical Ethics Committee of the Rehabilitation Foundation Limburg, the Netherlands.

### Instruments

For all participating patients:

#### Psychological distress

The Dutch Version of the Symptom Checklist (SCL-90) was used to assess psychological distress. The SCL-90 is a multidimensional state measure of psychopathology and consists of eight dimensions: anxiety, agoraphobia, depression, somatic symptoms, distrust and interpersonal sensitivity, anger hostility as well as sleeping disorders. The total SCL-90 score reflects general psychoneurotiscism or psychological distress. Reliability and validity of the Dutch version of the SCL-90 have been reported to be adequate [11,12].

#### Fear of movement/(re)injury

The Dutch version of the Tampa scale for kinesiophobia (TSK) measures fear of movement. This questionnaire contains 17 items and is aimed to assess fear of (re)injury due to movement. The Dutch version of the TSK has been reported to be reliable and valid [6,13,14].

In addition, for the patients with fibromyalgia the following instruments were assessed:

#### Disability

The Dutch version of the Fibromyalgia Impact Questionnaire (FIQ) was used to score disability due to fibromyalgia. The FIQ consists of 10 items. The scores of each item are standardized on a scale ranging from 0–10 with higher scores indicating a higher level of impairment. The FIQ is validated for the Dutch language and its reliability, construct validity and responsiveness appeared to be sufficient [15].

#### Health related quality of life

The SF36 is a generic instrument measuring health related quality of life [16]. It comprises 8 subscales: physical functioning, role limitations because of physical health, role limitations because of emotional health, mental health, social functioning, bodily pain, vitality and general health. All subscales range from 0 to 100, with a higher value indicating a better perceived health. The Dutch version of the SF36 was used to measure health related quality of life in the subgroup of patients with fibromyalgia [16,17].

### Statistical analysis

To answer the first research question, contributing factors for the explanation of disability in fibromyalgia were explored based on a linear regression analysis. The dependent variable in the regression model was disability as measured with the total FIQ score. Independent variables were selected based on a holistic view on pain related disability in which biomedical, psychological as well as social factors have their influence. From a biomedical perspective gender and physical functioning (subscale of the SF36) were included as independent variables. To reflect psychological functioning both fear of injury and mental health (subscale of the SF36) were included. A patient's social situation was represented by social functioning, which is assessed based on the score on this subscale of the SF36. Collinearity control included checking variable inflation factors (VIF), which had to be below 10. Extreme values, more than 3 box lengths from the upper or lower edge of the box, and outliers, with Cook's distance above 1, were discarded.

To answer the second research-question three groups of patients with different pain syndromes were compared using the following tests: (1) a χ^2 ^analysis for dichotomous variables; (2) a one-way analysis of variance (ANOVA) including a post hoc range test according Tukey for normal distributed continuous variables; (3) a Kruskal-Wallis one-way analysis of variance for non-normal distributed continuous variables (two tailed-test with significance level of p < 0.05).

To interpret the impact of the fibromyalgia syndrome on the quality of life, the total score of the SF36 of patients with fibromyalgia in this study was studied in comparison with scores of patients with fibromyalgia from other studies, quality of life scores of patients with other chronic pain conditions and scores of persons out of the general population.

Analyses were performed using SPSS software (SPSS Inc., Chicago, Ill. Version 14).

## Results

111 patients participated in this study: 54 patients with fibromyalgia, 22 patients with CRPS and 35 patients with CLBP. Of the patients with fibromyalgia, 33.3% was referred by their general practitioner, 58.3% by their rheumatologist and 8.3% by another medical specialist. Main patient characteristics are presented in Table 1. Both in patients with fibromyalgia and CRPS, significantly more women were represented in comparison with the gender-distribution within the group of CLBP-patients (p < 0.01). Median age didn't differ between the three groups. Median duration of complaints was 8 years for patients with fibromyalgia, 1.5 years for patients with CRPS and 9 years for patients with CLBP. This difference in pain-duration between the groups appeared to be significant (p < 0.01).

**Table 1:** Patient characteristics (N = 111)

	**Fibromyalgia (N = 54)**	**CRPS (N = 22)**	**CLCP (N = 35)**	**p-value**
Number of patients	54	22	35	
Gender (M/F)	7/47	5/17	20/15	<0.01
Age (years)	40.0 (32–48)	45.5 (34–52)	44.0 (37–50)	0.21
Duration of pain complaints (years)	8 (4–10)	1.5 (0.5–4)	9 (3–15)	<0.01

Median scores with a 25–75% interval are presented.

CRPS = Complex Regional Pain Syndrome; CLBP = Chronic Low Back Pain.

In view of answering the first research question in Table 2 disabling factors for patients with fibromyalgia are presented based on the results of the linear regression analysis. Mental health appeared to be the strongest contributor to the explained variance of disability with an exp β = -0.360 (p < 0.02). Physical functioning contributed significantly with an exp β = -0.290 (p = 0.05). Neither gender, nor fear of injury had a significant influence in the model. VIF factors were low (with a maximum of 1.8), indicating that there was no interfering interaction between the variables in the model. Cook's distances did not exceed 1 which indicated that no outliers were  present.

**Table 2:** Disability in fibromyalgia: linear regression analysis with disability as dependent variable (N = 54)

**Dependent variable**	**Independent variable**	**R^2^**	**Adj R^2^**	**Standardized β**	**p-value**
Disability	Gender	0.408	0.341	0.136	0.25
	Social functioning			-0.216	0.17
	Mental health			-0.360	0.02
	Physical functioning			-0.290	0.05
	Fear of injury			-0.101	0.45

In order to test the impact of the pain syndrome on mental health as a prominent disabling factor, psychological profiles of patients within the three groups of pain syndromes were further analysed. The psychological profiles of patients with fibromyalgia and other pain syndromes (CRPS and CLBP) are presented in Table 3. Since the distribution of scores for the total score of psychological distress including several subscales, were skewed, Kruskal Wallis testing was used. Median total score for psychological distress in patients with fibromyalgia was significantly higher as compared to scores of both other pain conditions (p < 0.01). In addition, the scores of the SCL-90 subscales phobic anxiety, depression, somatic symptoms, obsessive compulsive and hostility of the groups appeared to be significantly different; patients with fibromyalgia scored significantly higher as compared to both other groups, except for hostility. The results of the hostility subscale revealed that the score of the CLBP patients was significantly lower as compared to the scores for both fibromyalgia and CRPS patients. Although for phobic anxiety a significantly higher score for patients with fibromyalgia was found, this finding could not be confirmed for a specific anxiety disorder as fear of injury based on the TSK-score. Although median TSK representing fear of injury appeared to be slightly higher in both subgroups with CRPS (41.0 (36–46)) and CLBP (39.5 (36.5–45)) as compared to the fibromyalgia patients (38.2 (30–43.6), this difference was not significant as tested based on Kruskal Wallis testing (p = 0.20).

**Table 3:** Psychological distress in fibromyalgia, CRPS and CLBP

	**Fibromyalgia (N = 54)**	**CRPS (N = 22)**	**CLBP (N = 35)**	**p-value**
Total score psychological distress	192 (161–239)	159 (128–190)	152 (126–202)	<0.01
Agorofobia	9 (7–13)	8 (7–14)	9 (7–13)	0.96
Phobic anxiety	19 (15–26)	15 (13–21)	15 (12–22)	0.02
Depression	41 (29–50)	28 (23–34)	25 (20–40)	<0.01
Somatisation	36 (29–41)	29 (22–38)	27 (22–36)	<0.01
Obsessive compulsive	25 (20–31)	20 (16–25)	19 (17–25)	<0.01
Interpersonal sensitivity	31 (24–41)	28 (22–35)	26 (20–33)	0.07
Hostility	10 (8–12)	9 (6–11)	7 (6–8)	<0.01
Sleeping disorders	11 (7–13)	10 (6–12)	8 (5–12)	0.06

Median scores combined with a 25–75% interval are presented.

CRPS = Complex Regional Pain Syndrome; CLBP = Chronic Low Back Pain.

The health related quality of life of patients with fibromyalgia as related to the quality of life of patients in other chronic conditions is presented in Table 4. Results of quality of life of patients participating in the current study seem to be in accordance with scores of patients with fibromyalgia participating in other studies. For patients with fibromyalgia in the current study, seven of the eight subscales of the SF36 (except the subscale vitality) were significantly (and negatively) associated with their level of fibromyalgia related disability, indicating that the quality of life scores were indeed influenced by fibromyalgia. As compared to the general population, patients with fibromyalgia seem to experience a high impact on their quality of life. In patients with fibromyalgia the impact of the pain syndrome on social functioning and mental health as measured by the SF36, seems to exceed the impact of rheumatoid arthritis. However, since data were derived from other studies, no statistical testing could be performed.

**Table 4:** Quality of life in patients with fibromyalgia, other chronic pain conditions and the general population (SF36)

	**N**	**Population**	**Physical functioning**	**Role limitations because of physical health**	**Role limitations because of emotional health**	**Mental health**	**Social functoning**	**Bodily pain**	**Vitality**	**General health**
***Fibromyalgia*** Current study	54	visitors of a rehabilitation department	37.8 (18.0)	8.3 (19.4)	51.6 (45.0)	55.1 (18.7)	44.7 (22.3)	30.8 (15.8)	34.6 (18.7)	38.5 (20.1)
Martinez et al, 2001 [32]*	32	visitors of a reum. out-patient clinic	39.4 (5–85)	14.8 (0–75)	32.3 (0–100)	44.3 (12–90)	45.1 (2–100)	26.5 (10–61)	38.6 (5–85)	43.3 (10–77)
***Other pain*** *Reumatoid arthritis* Ruta et al, 1998 [33]	233	visitors of a reum. clinic	31 (29)	25 (38)	59 (42)	69 (20)	54 (33)	37 (23)	39 (24)	44 (23)
Aaronson et al, 1998 [34]	485	visitors of a internal med. department	63.3 (25.1)	35.0 (40.3)	58.4 (43.6)	68.0 (19.8)	73.9 (24.1)	69.3 (26.6)	60.1 (22.3)	52.5 (21.4)
*CLBP* Merkesdal et al, 2003 [35]	150	out patient rehabilitation patients	44 (20)	11 (17)	20 (24)	43 (12)	44 (20)	23 (13)	29 (11)	34 (12)
Tavafian et al, 2007 [36]	52	visitors of a reum. Centre	52.5 (20.2)	31.7 (35.0)	32.7 (40.4)	47.8 (23.5)	62.5 (29.8)	42.6 (25.3)	48.9 (21.6)	41.7 (22.2)
***General population*** Edlinger et al, 1998 [37]	4423	national Dutch survey	83.0 (22.9)	76.4 (36.3)	82.3 (32.9)	76.8 (17.4)	84.0 (22.4)	74.9 (23.4)	68.6 (19.3)	70.7 (20.7)

Mean scores (SD) are presented * the study of Martinez et al only median scores are presented.

CLBP: chronic low back pain

## Discussion

Based on the results of this study, the level of perceived disability in patients with fibromyalgia seemed best explained by their mental health condition and less by their physical condition. Furthermore, it appeared that the level of psychological distress was higher in patients with fibromyalgia as compared to patients with CRPS or CLBP. The impact of fibromyalgia on the quality of life appeared to be considerable.

### Shortcomings of the study

This study is performed based on data of patients entering rehabilitation departments in a chronological order. Although patients with fibromyalgia entered the study after referral by consultants in rehabilitation medicine of one of five rehabilitation departments, patients with CRPS and CLBP were included out of only one department. As a result, the number of patients in both other pain conditions (22 with CRPS and 33 with CLBP) is rather low, and group sizes are unequal. For this reason non-parametrical testing was used when population based scores were compared. The group size of patients with fibromyalgia also had implications for the regression analysis performed. Due to the number of 54 participants, the number of independent variables that could be introduced in the regression analysis was rather limited. As a result of this, only the most prominent factors out of the explanatory models for pain related disability were chosen and introduced in the current regression model. A second drawback within this study is the fact that the composition of the three groups of patients appeared to be unequal regarding the male/female ratio. In addition, although all patients had a chronic pain condition, patients with fibromyalgia and CLBP had pain for a longer period in comparison with CRPS patients. However, differences in pain duration will presumably not have influenced the results of this study. Although median pain duration of 1.5 years for patients with CRPS was significantly shorter, this time period seems however extensive enough to elicit psychological distress. On the other hand, an interfering influence of gender could be hypothesized. For this reason, in the regression analysis the model is corrected for gender. Based on the results of the regression analysis, it appeared that gender did not significantly influence the explanation of the level of disability. In the analysis for psychological distress no correction for gender was performed, merely because of the low number of participants. As a result of this, in interpreting the result of this analysis, gender related differences have to be considered. Thirdly, the inclusion of the three groups of patients with chronic pain was based on their referral to secondary or third care rehabilitation care. Patients who entered the study visited the department of a rehabilitation specialist referred by different medical specialists or general practitioners. For the patients with fibromyalgia, at the moment of inclusion in the study, no additional check, such as a check of the American College of Rheumatology guideline, was performed by the researcher or consultant in rehabilitation medicine. However, before entering the rehabilitation department, for 94.6% of the patients fibromyalgia was diagnosed by a rheumatologist, according to their professional reumatological guidelines, which includes a check of the criteria of the American college of Rheumatology. In case the analyses were repeated with patients included referred by the rheumatologist (N = 51), results didn't differ from the results found on the total population of patients with fibromyalgia. The study population represents a population of patients with fibromyalgia and CRPS normally being referred to secondary care rehabilitation services in The Netherlands. The CLBP patients however, were referred to a tertiary rehabilitation centre, which might indicate that their level of distress and fear of injury might be higher than the ones normally seen in secondary care.

### Psychological distress

In this study, it appeared that the level of psychological distress of patients with fibromyalgia exceeds the scores for patients with CRPS and CLBP. The median score of 159 and 152 for consecutively patients with CRPS and CLBP are in agreement with published mean score of 146 (SD 49) for male and 150 (SD 44) for female patients with chronic pain who visited Dutch pain clinics N = 2458 [12]. In contrast, patients with fibromyalgia score higher. The finding that scores for somatisation, depression and anxiety are higher in patients with fibromyalgia as compared to scores for patients with other pain conditions have been reported before. Most studies addressing psychological distress in patients with fibromyalgia compared "fibromyalgia scores" with scores of patients with rheumatoid arthritis and reported somatisation rates for patients with fibromyalgia that exceed those found in patients with rheumatoid arthritis [18-20]. In addition, the level of anxiety in patients with fibromyalgia appeared to be higher as compared to the level of anxiety in patients with rheumatoid arthritis [20-22]. Lifetime depression rates in fibromyalgia ranged from 20% to even 86% indicating a high prevalence in comparison with other medical conditions [23,24]. Raphael et al, reported that in women with fibromyalgia the risk of lifetime anxiety disorders and in particular obsessive compulsive disorder, appeared to be approximately 5-fold higher as compared to the general population [25].

### Disability in fibromyalgia

The fact that depression and anxiety appeared to be high in patients with fibromyalgia could support the hypothesis of van den Houdenhoven that in patients being deprived of "overactivity" as their favourite coping strategy, anxiety and depression can occur and can have a substantial influence on the level of disability. The fact that in the regression-analysis, mental health contributed significantly in the explanation of a patient's disability level confirms the influence of psychological wellbeing on the functioning of patients with fibromyalgia. It is of great importance to identify factors that are associated with disability in patients who persist in performing activities, as this group may be distinguished from patients who use avoidance strategies to cope. The idea of van Houdenhoven regarding disability in chronic pain patients with a premorbid overactive lifestyle matches the ideas explained in the self-discrepancy model of Higgins [26]. In view of the explanation of disability in fibromyalgia, the selfdiscrepancy model is introduced in the discussion section of this article as a suggestion for further research. Higgin's self discrepancy theory postulates that each person has three basic domains of selves; the actual selve (e.g. describes what attributes an individual believes they actually possess), the ideal selve (the characteristics that an individual would ideally like to possess in the future) and the ought selve (the attributes that an individual believes they ought to or should possess). Individuals are motivated to work towards a condition where the actual self matches the ideal self or ought self. In this, people strive to keep the discrepancies between the actual-ideal and the actual-ought selves as small as possible, as these give rise to negative psychological situations, that are associated with specific emotions [27]. This could be the explanation of persistent behaviour as reported by van Houdenhoven and Hasenbring. According to Higgins, discrepancies between actual-ideal self gives rise to dejection-related emotions (e.g. disappointment, frustration, depression), while a discrepancy between the actual-ought self may lead to agitation related emotions (e.g. fear, guilt, self-contempt). This theory postulates that discrepancies between selves (actual self vs. ideal self and actual self vs. ought self) gives rise to specific negative emotions, which will finally lead to disability. According to the self-discrepancy theory, the greater the magnitude and accessibility of a particular type of self-discrepancy, the higher the intensity of the associated discomfort when that particular self-discrepancy is activated. The concept of self-discrepancy has been applied to a number of clinical disorders, such as body dysmorphic disorder [28], depression and anxiety [29]. Davies was the first to apply the self-discrepancy theory and its concept of self and identity to a group of chronic pain patients [30]. She found in 89 patients with different types of specific and nonspecific pain that self-discrepancies are significant predictors for depression, anxiety and pain-related disability. In our study based on the presented data, no conclusions can be drawn on the role of the concept of selfdiscrepancies in fibromyalgia. However, the fact that psychological factors as depression and general anxiety were high in patients with fibromyalgia together with the fact that mental health was most associated with disability could support the idea of the self discrepancy theory. The finding of Natvig et al that patients with fibromyalgia had a higher leisure time physical activity level as compared to other females without pain [31] could confirm the hypothesis on the disabling role of persistent behaviour in fibromyalgia. Further research is warranted.

### Quality of life

It seems that patients with fibromyalgia experience a lower quality of life as compared to the general population. In comparison with patients with rheumatoid arthritis, especially mental health and social functioning of patients with fibromyalgia seem to be more affected. Quality of life of patients with fibromyalgia was associated with their fibromyalgia related disability level. The total impact on quality of life of fibromyalgia, as in CLBP, seem considerable. However, in comparing data of the different studies, it is important to consider that: Firstly, no statistical testing has been performed to confirm differences in scores on quality of life of the different patients groups and secondly data within Table 4, are gathered from both patients who searched for help in a rheumatologic or rehabilitation department together with persons who filled in a questionnaire based on a survey or an advertisement and were not seeking medical care. As a result of these different recruitment procedures, differences between populations regarding the level of quality of life may have occurred. Therefore, in the interpretation of these results on quality of life, the risk of selection bias has to be considered. Nevertheless, based on this overview, it can be concluded that the quality of life of patients with fibromyalgia seem to be influenced by their pain problem.

### Implications for clinical practice and further research

If it can be confirmed that in a population of patients with fibromyalgia, especially patients with persistent behaviour seem to be present, these patients will not benefit from the current approach in rehabilitation medicine focusing on enhancing the level of physical activity. It may be speculated that patients who show persistent behaviour might benefit from learning how they can reduce for example their self discrepancies and associated negative emotions and fine tune their activities during the day. Distinguishing groups of activity related behaviour seems therefore an important research topic. This could lead to selecting specific treatments in the future for different patients with chronic pain, and especially in patients with fibromyalgia.

This study is based on a cross sectional design and hypotheses on disabling factors for fibromyalgia are given. To study contributing factors in chronification of the pain related syndromes and their impact on daily life a prospective cohort study seems more appropriate as compared to the current cross sectional design. However, we believe that the preliminary results of the current study are of value for further research. As a result of this, further research is warranted.

## Conclusion

Based on the results of this study, it can be concluded that patients with fibromyalgia report a high impact on their quality of life. The level of perceived disability in patients with fibromyalgia seemed best explained by their mental health condition. It appeared that the level of psychological distress was higher in patients with fibromyalgia as compared to patients with other pain syndromes.

## Abbreviations

ANOVA: One-way Analysis of Variance; CLBP: Chronic Low Back Pain; CRPS: Chronic Regional Pain Syndrome; FIQ: Fibromyalgia Impact Questionnaire; SCL90: Symptom Checklist; SD: Standard Deviation; SF36: 36 Items Short Form Health Survey; SPSS: Statistical Package for the Social Sciences; TSK: Tampa Scale of Kinesiophobia; VIF: Variable inflation factor.

## Competing interests

The author(s) declare that they have no competing interests.

## Authors' contributions

JAV was the first author of this study and had an initiating role in all phases of the study. DHP had an important role in the acquisition of the data and had a substantial contribution in drafting the manuscript. RJS has been involved in the analysis and interpretation of the data in combination with drafting the manuscript. All authors read and approved the final manuscript.
